# Targeting digestive system cancers with isoliquiritigenin: a comprehensive review of antitumor mechanisms

**DOI:** 10.3389/fphar.2025.1649472

**Published:** 2025-09-04

**Authors:** Zhichun Li, Ruohan Feng, Jinze Li, Jinglu Bai, Nuo Li, Zhenhua Cui, Xiaobin Zhang

**Affiliations:** 1 Medical College, Shandong University of Traditional Chinese Medicine, Jinan, Shandong, China; 2 College of Pharmacy, Shandong University of Traditional Chinese Medicine, Jinan, Shandong, China; 3 College of Traditional Chinese Medicine, Shandong University of Traditional Chinese Medicine, Jinan, Shandong, China; 4 Department of Colorectal and Anal Surgery, The Second Hospital of Shandong University, Jinan, Shandong, China; 5 Department of Traditional Chinese Medicine External Treatment Center, Affiliated Hospital of Shandong University of Traditional Chinese Medicine, Jinan, Shandong, China

**Keywords:** isoliquiritigenin, digestive system cancers, licorice, mechanism, natural compounds

## Abstract

Digestive system malignancies, including gastric, colorectal, and liver cancers, account for a substantial proportion of global cancer morbidity and mortality. Despite advancements in conventional treatment strategies such as chemotherapy, radiotherapy, surgical resection, and immunotherapy, clinical outcomes remain unsatisfactory due to limited therapeutic efficacy, severe side effects, and poor patient prognosis. Therefore, the development of more effective and less toxic treatment options is urgently needed. Isoliquiritigenin (ISL), a natural chalcone-type flavonoid primarily extracted from licorice (*Glycyrrhiza* spp.), has attracted increasing attention for its broad pharmacological properties, including anti-inflammatory, antioxidant, antiviral, and anticancer activities. Recent studies have revealed the potential of ISL to modulate multiple signaling pathways and cellular processes involved in tumorigenesis, such as apoptosis, autophagy, ferroptosis, cell cycle regulation, and immune modulation. This review comprehensively summarizes the current understanding of ISL’s anticancer mechanisms in digestive system tumors, highlighting its multi-targeted actions and potential as a promising therapeutic agent. The findings aim to provide valuable insights for future preclinical studies and clinical applications.

## 1 Introduction

According to GLOBOCAN 2020 statistics, digestive system malignancies rank among the leading causes of global cancer incidence and mortality. Gastric cancer, colorectal cancer, and liver cancer collectively account for 20.1% of newly diagnosed cancer cases and 25.2% of cancer-related deaths worldwide (Sung et al., 2021). The high mortality rates of digestive system tumors across countries at varying stages of development are largely attributed to poor dietary habits, alcohol consumption, tobacco use, and the inherently aggressive nature of these malignancies, which are often associated with high incidence, rapid progression, and poor clinical prognosis (Sung et al., 2021; Habal et al., 2000; Torre et al., 2016). With the advancement of medical technologies, various treatment strategies have been developed for digestive system tumors, including chemotherapy, radiotherapy (Allemani et al., 2018), surgical resection, immunotherapy (Mikhail and Bekaii-Saab, 2015; Song et al., 2017), and organ transplantation (Habal et al., 2000; Zhang et al., 2017; Erstad and Tanabe, 2019; Slevin et al., 2022). However, these modalities are limited by narrow therapeutic scopes (Song et al., 2017; Lamarca et al., 2020), suboptimal efficacy (Warnakulasuriya, 2009), invasive surgical procedures, postoperative metastasis, and the severe adverse effects of radiotherapy and chemotherapy which significantly impair patients’ quality of life (Yang et al., 2016). Consequently, there is an urgent need to develop more effective and less toxic therapeutic agents for the treatment of digestive system cancers.

Plant-derived bioactive compounds are known for their anticancer properties, characterized by low toxicity and high efficacy (Lin et al., 2019; Zhang et al., 2024). Licorice, a traditional Chinese medicinal herb, has been widely studied for its antitumor activities, which are believed to be mediated through the suppression of cytokine levels, interference with the cell cycle, and induction of cancer cell apoptosis. Licorice contains a variety of flavonoids and triterpenoids (Yang et al., 2015), including 11-deoxoglycyrrhetinic acid (11-DOGA) (Lin et al., 2014), glycyrrhetinic acid (GA) (Kim et al., 2013), glycyrrhizic acid (GLD) (Hsu et al., 2011), licochalcone A (LCA) (Kim et al., 2010; Szliszka et al., 2012), and licochalcone B (LCB) (Yuan et al., 2014).

**FIGURE 1:**
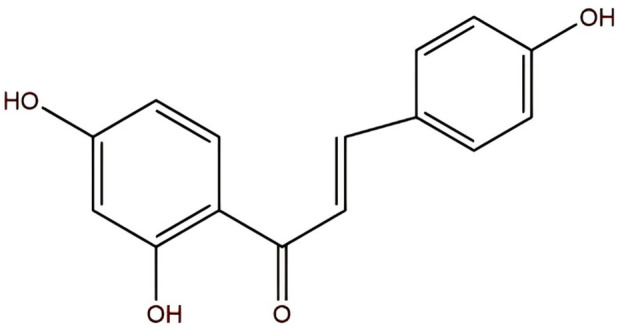
The chemical formula of ISL is C_15_H_12_O_4_.

ISL, with the molecular formula C_15_H_12_O_4_, is chemically known as (E)-1-(2,4-dihydroxyphenyl)-3-(4-hydroxyphenyl)-2-propen-1-one or 4,2′,4′-trihydroxychalcone (Figure 1). It appears as yellow needle-like crystals with a melting point of 198 °C–200 °C. Currently, ISL is primarily isolated from licorice (Glycyrrhiza spp.) (Zhao et al., 2019), and various extraction techniques have been developed, including acid hydrolysis (Yujie et al., 2007), supercritical CO_2_ extraction (Yujie et al., 2003), and purification using macroporous adsorption resin (Yujie et al., 2006). ISL exhibits a wide range of pharmacological activities, including anti-inflammatory (Gu et al., 2020), antiviral (Traboulsi et al., 2015), antioxidant (Zhu et al., 2019), and anticancer effects (Zhao et al., 2019). Studies have revealed that ISL exerts therapeutic effects in prostate cancer (Zhang et al., 2010), renal carcinoma (Yamazaki et al., 2002), several types of gynecological malignancies (Wang et al., 2013; Lin et al., 2020; Wang et al., 2014; Wang et al., 2015), and respiratory system tumors (Tian et al., 2018; Jung et al., 2014; Chen et al., 2018; Cui et al., 2022), exert anti-tumor effects through multiple targets and multiple pathways (Zhang et al., 2022a). This review summarizes the biological mechanisms by which ISL regulates digestive system tumors, aiming to provide new insights and strategies for basic research and clinical applications.

## 2 Mechanistic insights into the antitumor effects of ISL in digestive system cancers

ISL has demonstrated potent anticancer activity against a variety of digestive system malignancies through modulation of multiple molecular pathways. These effects are mediated via the regulation of apoptosis, autophagy, cell cycle arrest, redox homeostasis, and tumor microenvironment (TME) remodeling. The following sections provide a detailed overview of the specific mechanisms by which ISL exerts its therapeutic effects in distinct types of digestive system cancers, including oral, gastric, colorectal, hepatic, gallbladder, and pancreatic cancers (Table 1).

**TABLE 1:** Therapeutic potential of ISL in digestive system malignancies.

Cancer	Possible mechanisms	Real modules (animal/cell)	Targets	Doses	References
Oral Cancer	Apoptosis, Cell Cycle Arrest	CAL27/SCC4 cells; Nude mice	Survivin ↓, p-Akt ↓, p-CDK1 (Thr161) ↓, p-CDK1 (Tyr15) ↑, p-Wee1↓, cleaved caspase-3 ↑, G2/M	*In vitro*: 20–100 μM; *In vivo*: 10–30 mg/kg	Zhou et al. (2023)
	Chemoresistance Reversal, Cell Cycle Arrest	SAS/OECM-1 CSCs; Nude mice	GRP78 ↓, ALDH1 ↓, CD44 ↓, ABCG2 ↓, PI3K, AKT, MAPK, G1/S	*In vitro*: 25–400 μM; *In vivo*: 5 mg/kg	Hu et al. (2017)
	Apoptosis, Cell Cycle Arrest	Tca8113 cells	Bcl-2 ↓, Bax ↑, MMP-2/9 ↓, Cyt C, caspase-3/7 ↑, G1/S	*In vitro*: 6.25–50 µM	Hou et al. (2017)
	Apoptosis, Cell Cycle Arrest	HSC-3,OEC-M1,SAS,Ca9-22,SCC4 cells; Nude mice	γ-H2A.X ↑, pATM ↓, pATR ↓, Mre11 ↓, Rad50 ↓, Nbs1, pChk1 ↓, pChk2, pCdc25c ↓, CDK1/2 ↓, Cyclin A ↓, Cyclin B ↑, p53 ↑, p21 ↑, caspase-3 ↑, PARP ↑, G2/M	*In vitro*: 1.25–200 μM; *In vivo*: 5 mg/kg	Hsia et al. (2016)
Gastric Cancer	Apoptosis, Cell Cycle Arrest	SGC-7901, BGC-823 cells	G2/M, Cdc2 ↓, Cyclin B1 ↓, Cleaved-PARP ↑, Bcl-2 ↓, Bax ↑, LC3B ↑, Beclin 1 ↑, p62 ↓, PI3K, AKT, mTOR, p-AKT ↓, p-mTOR ↓	20–40 μM	Huang et al. (2020a)
	Apoptosis, Self-renewal ability, Tumor Microenvironment Modulation	MKN45 cells; Nude mice (MKN45 xenograft)	CREB3L1 ↓, GRP78 ↓, CD24 ↓, CD44 ↓, LGR5 ↓, SOX2 ↓, Nanog ↓, TGF-β1 ↓, IL-6 ↓, CAFs ↓, α-SMA ↓, MMP-9 ↓, ECM ↓, EMT ↓	*In vitro*: 15–25 μg/mL *In vivo*: 5-FU: 10 mg/kg (intraperitoneal)	Lee et al. (2022)
	Apoptosis, Cell Cycle Arrest	MKN28 cells	PI3K, AKT, mTOR, p-AKT ↓, p-mTOR ↓, Bcl-2 ↓, Bax ↑, Cleaved caspase-3 ↑, LC3Ⅱ/LC3Ⅰ ↑, Beclin 1 ↑, p62 ↑	20 μM	Zhang et al. (2018)
	Apoptosis, Cell Cycle Arrest	MGC-803 cells	Intracellular free calcium concentration ↑, mitochon, drial transmembrane potential (Δψ_m_) ↓	0.075–0.15 g·L^−1^	Ma et al. (2001)
	Proliferation, Metastasis Inhibition	MGC803, SGC7901 cells; Nude mice	GLUT4 ↓, PDHK1 ↓, PGC-1α↓, c-Myc ↓, HIF-1α↓, LDHA ↓, Bcl-2 ↓, Bax ↑, Cleaved caspase-3 ↑, Cleaved caspase-9 ↑	*In vitro*: 40–50 μM *In vivo*: 10–100 mg/kg	Yu et al. (2023)
Colorectal Cancer	Apoptosis, Cell Cycle Arrest	SW480, HCT116 cells; Nude mice	ESR2 ↑, PIK3CG ↓, GSK3β, p-AKT ↓, p-GSK3β ↓, CDK1 ↓, NF-κB ↓, Bcl-2 ↓, Bax ↑	*In vitro*: 20–140 μM; *In vivo*: 30–60 mg/kg	Luo et al. (2023)
	Chemoresistance Reversal	HCT-116, HT-29, SW-480 cells; Nude mice	p62/SQSTM1 ↑, caspase-8 ↑, cleaved PARP ↑, Fas ligand ↑	*In vitro*: 10–40 μM; *In vivo*: 2.5 mg/kg	Jin et al. (2018)
	Macrophage Polarization, Tumor Microenvironment Modulation	RAW264.7 cells, mouse peritoneal macrophages; Balb/c mice	PGE_2_ ↓, IL-6 ↓, COX- 2 ↓, STAT3 ↓, PPARδ ↓, EP4 ↓, iNOS ↑, arginase - 1 ↓, CD206 ↓	*In vitro*: 5–20 μM; *In vivo*: 3, 15, 75 mg/kg	Zhao et al. (2014)
	Gut Microbiota Modulation	BALB/c mice	Butyrate producers (Butyricicoccus, *Clostridium*) ↑; pathogens (*Escherichia*, *Enterococcus*) ↓; F/B ratio normalization ↓	150 mg/kg	Wu et al. (2016a)
	Proliferation, Metastasis Inhibition	HCT116, SW620, HT29 cells	AMPK ↑, p-mTOR ↓, ENO1 ↓, ALDOA ↓, LDHA ↓, MCT4 ↓, c-myc ↓, HIF-1α↓, p-Akt ↓, CAV1 ↓	25–100 μM	Wang et al. (2020)
Liver Cancer	Apoptosis, Cell Cycle Arrest, Autophagy	MHCC97-H, SMMC7721 cells; Nude mice	PI3K, p-PI3K ↓, AKT, p-AKT ↓, mTOR, p-mTOR ↓, LC3-II ↑, Beclin 1 ↑, P62 ↑, Bcl-2 ↓, Bax ↑, Cleaved caspase-3 ↑, Cleaved PARP ↑	*In vitro*: 12.5–50 μM *In vivo*: 50 mg/kg	Song et al. (2020)
	Apoptosis, Cell Cycle Arrest	HepG2, Hep3B cells; L-02, QSG-7701 normal liver cells	ROS ↑, JNK ↑, p38 ↑, ERK ↓, STAT3 ↓, NF-κB ↓, IκB ↑, Bcl-2 ↓, Bax ↑, Cleaved caspase-3 ↑, Cleaved PARP ↑, p21 ↑, p27 ↓, Cyclin B1 ↓, CDK1/2 ↓	*In vitro*: 20–100 μM (cell viability assay); 28 μM (apoptosis/cell cycle analysis)	Wang et al. (2019)
	Redox imbalance, Radiosensitization	HepG2 cells; Nude mice	Keap1 ↑, Nrf2 ↓, HO-1 ↓, NQO1 ↓, ROS ↑, γ-H2AX ↑, Rad51 ↑	*In vitro*: 10 μg/mL *In vivo*: 10 mg/kg	Sun et al. (2015)
Gallbladder Cancer	Apoptosis & Cell Cycle Arrest	NOZ, SGC996 cells; Human intrahepatic biliary epithelial cells (HiBECs), primary gallbladder epithelial L-2F7 cells; Nude mice	HMOX1 ↑, GPX4 ↓, p62 ↑, Keap1 ↓, p-Nrf2 ↑, ROS ↑, Fe^2+^ ↑, lipid peroxides ↑, GSH/GSSG ↓	*In vitro*: 20–100 μmol/L; *In vivo*: 50 mg/kg	Wang et al. (2023b)
Pancreatic Cancer	Proliferation, Metastasis Inhibition	PANC1, MIA PaCa-2 cells; Pan02 cells; Nude mice	p38 MAPK, p-p38 ↑, LC3II/LC3I ↑, p62 ↑, Atg5 ↓, Cleaved PARP ↑, γH2AX ↑	*In vitro*: 12.5–25 μM; *In vivo*: 30–60 mg/kg	Zhang et al. (2022c)

### 2.1 Anticancer mechanisms of ISL in oral cancer

Oral cancer is a common malignancy ranked eighth in global cancer prevalence and is primarily caused by tobacco use, alcohol consumption, betel nut chewing, and infection with human papillomavirus (HPV) (Rapidis et al., 2009; Wang Y. et al., 2023). It typically occurs on the lips, tongue, and floor of the mouth, with squamous cell carcinoma (SCC) accounting for over 90% of cases (Oj et al., 2012). Several molecular factors regulate the progression of oral cancer. For instance, survivin plays a role in cell division and apoptosis inhibition (Yesupatham et al., 2023; Ju et al., 2017), while glucose-regulated protein 78 (GRP78) mediates tumor metastasis (Lizardo et al., 2016), proliferation (Yin et al., 2017), and resistance to chemotherapy and radiotherapy (Gu et al., 2015).

To suppress tumor growth and overcome drug resistance, ISL has been shown to exert antitumor effects by inhibiting the Akt–Wee1–CDK1 signaling pathway. This inhibition reduces phosphorylation of survivin at the Thr34 residue, facilitating its ubiquitin-mediated degradation. Given survivin’s pivotal role in tumor cell survival, its degradation weakens the viability of cancer cells and suppresses tumor growth in CAL-27 oral squamous carcinoma models. Furthermore, this mechanism helps overcome cisplatin resistance in oral SCC, offering a promising strategy for enhancing chemotherapy efficacy (Zhou et al., 2023).

In addition, Hu et al. demonstrated that ISL downregulates drug resistance–related ABC transporters. Thereby impairing the self-renewal and invasiveness of oral squamous cell carcinoma cancer stem cells (OSCC-CSCs). These CSCs, known for their self-renewal capacity and aggressive behavior, are key drivers of tumor recurrence and metastasis. ISL exerts its effects by disrupting the niche of CSCs, thereby inhibiting tumor invasion. Meanwhile, when combined with cisplatin, it significantly suppresses the self-renewal of OSCC-CSCs, reverses T-cell exhaustion, and synergizes with chemotherapy to enhance immune responses. ISL’s ability to suppress these characteristics not only attenuates tumor malignancy but also enhances chemosensitivity, allowing anticancer drugs to more effectively target tumor cells and improve therapeutic outcomes (Hu et al., 2017).

S- ISL, a synthetic derivative of ISL, is produced via a Mannich reaction that introduces nitrogen-containing heterocycles or open-chain amino compounds into the A-ring side chain of ISL (Fu et al., 2014; Zhang et al., 2008). S-ISL has demonstrated broad-spectrum inhibitory effects on Tca8113 oral cancer cells, significantly suppressing proliferation, reducing adhesion, and impairing migration and invasion, while promoting apoptosis. Mechanistically, S-ISL exerts its antitumor effects by modulating the expression of apoptosis- and metastasis-related proteins: it downregulates the anti-apoptotic protein B-cell lymphoma 2 (Bcl-2) and upregulates the pro-apoptotic protein Bcl-2-associated X protein (Bax), enhancing apoptotic signaling. Additionally, it inhibits matrix metalloproteinases Matrix Metalloproteinase-2 (MMP-2) and Matrix Metalloproteinase-9 (MMP-9), reducing invasiveness, and decreases reactive oxygen species (ROS) production, thereby limiting oxidative stress–induced tumor progression (Hou et al., 2017). It should be clarified that the mechanism by which ISL exerts its effects in oral tumors is unique, specifically manifested as follows: directly impairing the survival ability of tumor cells by inhibiting the Akt-Wee1-CDK1 signaling pathway and promoting the ubiquitin-mediated degradation of Survivin protein; overcoming cisplatin resistance by downregulating ABCG2/GRP78; and the therapeutic effect of S-ISL.

**FIGURE 2:**
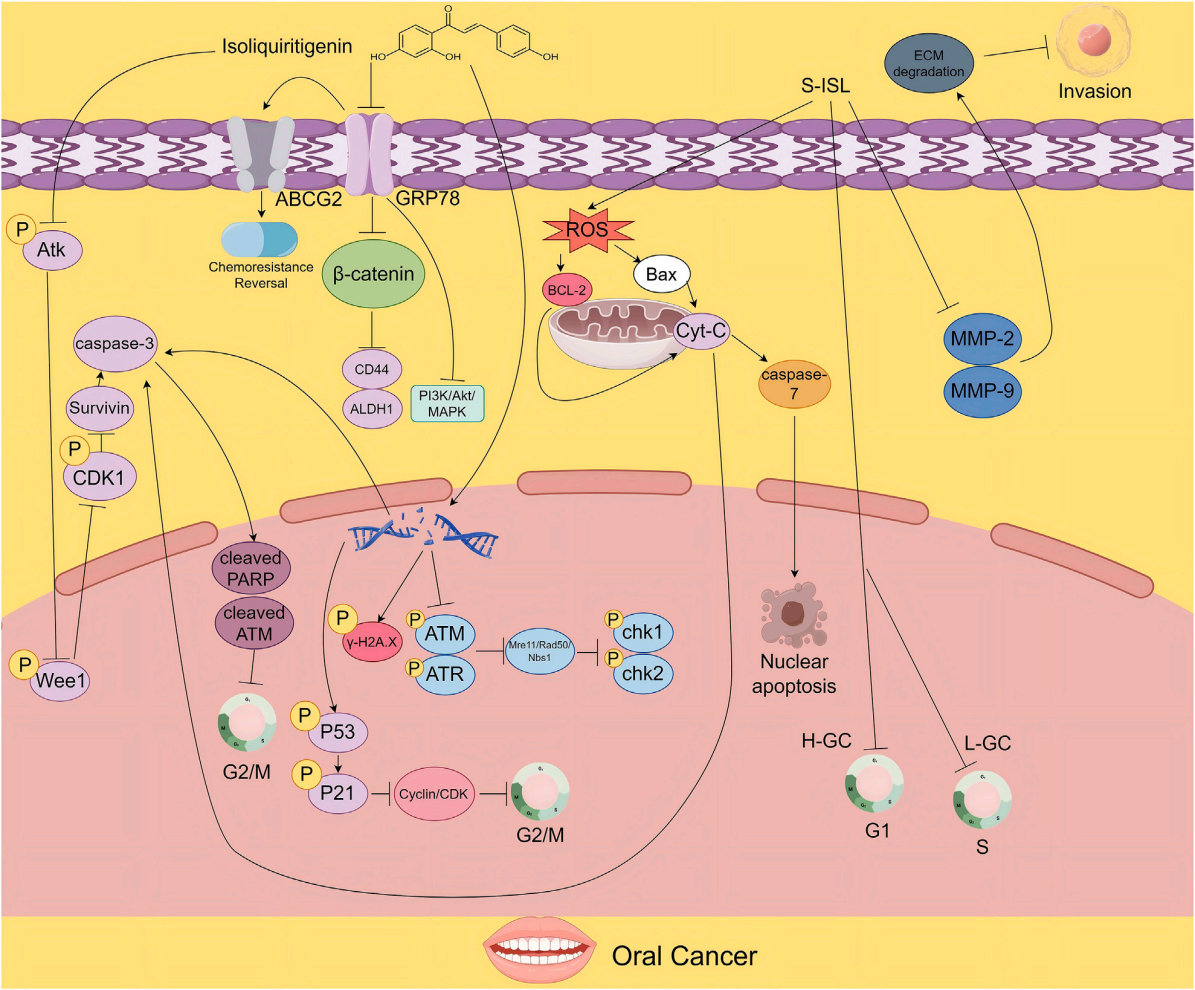
The figure illustrates the proposed mechanisms by which ISL exerts its antitumor effects in oral cancer. ISL promotes survivin degradation by inhibiting the Akt-Wee1-CDK1 signaling pathway. It also suppresses tumor growth and enhances chemosensitivity by downregulating drug resistance - associated proteins. Moreover, its derivative S-ISL exhibits antitumor activity by modulating apoptosis- and metastasis-related proteins, including Bcl-2 and Bax, and by reducing ROS production. In addition, ISL can disrupt DNA repair mechanisms, inducing cell cycle arrest and apoptosis, thereby inhibiting tumor proliferation.

In terms of DNA repair and cell cycle regulation, ISL activates caspase-mediated cleavage of ataxia telangiectasia mutated (ATM) protein, thereby impairing DNA repair mechanisms in OSCC cells. The resulting accumulation of DNA damage induces G2/M cell cycle arrest, halting cell division and ultimately triggering apoptosis, effectively controlling tumor cell proliferation (Hsia et al., 2016) (Figure 2).

### 2.2 Anticancer mechanisms of ISL in gastric cancer

Gastric cancer is the fourth most common malignancy worldwide (Ferlay et al., 2010; Lin et al., 2015) and the second leading cause of cancer-related mortality (Torre et al., 2015). Due to the lack of specific symptoms in early stages, most patients are diagnosed at advanced stages, resulting in poor prognosis. ISL and its novel analog ISL-17 have shown significant therapeutic potential against gastric cancer. ISL-17 suppresses tumor cell proliferation by inducing cell cycle arrest and apoptosis, increasing ROS levels, and enhancing autophagic activity. ISL also modulates the TME by downregulating GRP78 and inhibiting the PI3K/AKT/mTOR signaling pathway, thereby promoting apoptosis and autophagy while disrupting energy metabolism and restraining cancer cell growth.

ISL-17 is a newly synthesized analog of ISL designed to improve its pharmacological properties. Huang et al. introduced fluorine atoms into the ISL structure and synthesized 18 analogs, among which ISL-17 was identified as a promising candidate. Their study demonstrated that ISL-17 induced G2/M phase cell cycle arrest and apoptosis in gastric cancer cell lines. It also significantly elevated intracellular ROS levels and autophagic activity, thereby effectively inhibiting tumor cell proliferation and survival (Huang F. et al., 2020). These findings underscore the multi-target antitumor effects of ISL and its derivatives, suggesting their strong potential for both research and clinical applications in gastric cancer therapy.

Furthermore, Lee et al. demonstrated that ISL inhibits the self-renewal capacity of gastric cancer stem cells and the expression of related proteins by downregulating the expression of GRP78. Additionally, it blocks the activation of cancer-associated fibroblasts (CAFs), thereby preventing CAF-mediated stromal remodeling, regulating the TME, and suppressing tumor growth. Furthermore, ISL indirectly promotes the infiltration of immune cells and reverses the immunosuppressive microenvironment. This effect has also been validated in xenograft animal models (Lee et al., 2022).

At the molecular level, Zhang et al. reported that ISL inhibits the PI3K/AKT/mTOR signaling pathway, thereby promoting apoptosis and autophagy in MKN28 gastric cancer cells and reducing their proliferation, migration, and invasiveness (Zhang et al., 2018). Similarly, Ma et al. revealed that ISL induces apoptosis in MGC-803 cells through calcium- and actin-dependent mechanisms, as evidenced by increased intracellular calcium levels and decreased mitochondrial membrane potential (ΔΨm), in a dose-dependent manner (Ma et al., 2001).

**FIGURE 3:**
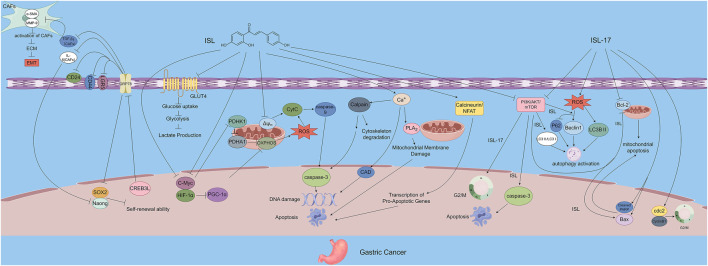
The figure illustrates the mechanisms by which ISL exerts its antitumor effects in gastric cancer. ISL-17, a synthetic derivative of ISL, inhibits tumor proliferation by inducing G2/M cell cycle arrest, promoting apoptosis, increasing ROS production, and enhancing autophagy. ISL downregulates GRP78 expression and inhibits the PI3K/AKT/mTOR signaling pathway, thereby modulating the TME, suppressing the self-renewal capacity of gastric cancer stem cells, and inactivating CAFs. In addition, ISL impairs energy metabolism by inhibiting GLUT4-mediated glucose uptake and interfering with both OXPHOS and glycolysis. Through the PDHK1/PGC-1α axis, ISL induces energy collapse and ROS accumulation, highlighting its multi-targeted antitumor potential.

Additionally, Yu et al. demonstrated that ISL inhibits GLUT4-mediated glucose uptake, reduces lactate production and secretion, and suppresses both mitochondrial oxidative phosphorylation (OXPHOS) and glycolysis. These effects lead to enhanced ROS accumulation and induction of energy collapse via the PDHK1/PGC-1α axis, ultimately suppressing gastric cancer cell growth. Notably, overexpression of Peroxisome Proliferator-Activated Receptor Gamma Coactivator 1α (PGC-1α), cellular Myc (c-Myc), Hypoxia-Inducible Factor-1α (HIF-1α), Glucose Transporter 4 (GLUT4), or Pyruvate Dehydrogenase Kinase 1 (PDHK1) was able to reverse the ISL-induced growth inhibition, confirming the central role of metabolic regulation in ISL’s antitumor activity (Yu et al., 2023). It can be concluded from the research results that, for gastric cancer among digestive system tumors, the therapeutic effects of ISL mediated by the energy targets GLUT4/PDHK1 and ISL-17 are its unique manifestations that distinguish it from other digestive system tumors (Figure 3).

### 2.3 Anticancer mechanisms of ISL in colorectal cancer

Colorectal cancer (CRC), a highly prevalent malignancy of the digestive system, ranks among the leading causes of cancer-related mortality worldwide (Sears and Garrett, 2014). The progression of CRC involves the synergistic regulation of various molecular mechanisms. For example, the PI3K/AKT signaling pathway plays a pivotal role in CRC cell proliferation and migration (Maharati and Moghbeli, 2023), while AMPK suppresses lactate production in CRC cells (Xiao et al., 2017). ISL has demonstrated multifaceted antitumor effects against CRC, involving the modulation of inflammation, signaling pathways, metabolic reprogramming, gut microbiota, and apoptosis.

Luo et al. reported that ISL activates the ESR2/PI3K/AKT signaling axis, thereby modulating the expression of several key proteins. Specifically, ISL downregulates Phosphatidylinositol-4,5-Bisphosphate 3-Kinase Catalytic Subunit Gamma (PIK3CG), AKT (Protein Kinase B), phosphorylated AKT (p-AKT), phosphorylated Glycogen Synthase Kinase 3β (p-GSK3β), Cyclin-Dependent Kinase 1 (CDK1), Nuclear Factor kappa-light-chain-enhancer of activated B cells (NF-κB), and Bcl-2, while upregulating Estrogen Receptor 2 (ESR2) and Bax. These changes reduce the p-AKT/AKT and p-GSK3β/GSK3β ratios and significantly increase the Bax/Bcl-2 ratio, resulting in suppressed CRC cell proliferation and enhanced apoptosis (Luo et al., 2023).

Jin et al. further demonstrated that ISL increases the expression of death receptor 5 (DR5), a receptor for tumor necrosis factor-related apoptosis-inducing ligand (TRAIL). This enhances the binding of TRAIL to DR5 and activates caspase-dependent apoptotic pathways, inducing CRC cell apoptosis. Additionally, ISL upregulates p62/SQSTM1, which modulates the activation of caspase-8 and regulates the apoptotic potential of CRC cells. Importantly, ISL acts synergistically with chemotherapeutic agents, enhancing their antitumor efficacy (Jin et al., 2018). In terms of regulating inflammation, Zhao et al. found that ISL downregulates the expression of prostaglandin E2 (PGE2) and interleukin-6 (IL-6), thereby blocking M2 macrophage polarization mediated by the PGE2/PPARδ and IL-6/STAT3 signaling pathways. This indirectly inhibits the activation of CAFs and collagen deposition, improves matrix stiffness, and effectively suppresses colitis-associated tumorigenesis through regulating macrophage-matrix interactions. Furthermore, this effect significantly reduces the activity of pro-tumor immune cells in the TME, decreases the secretion of immunosuppressive factors such as TGF-β and IL-10, relieves the inhibition of T cells, and reverses the immunosuppressive microenvironment. These findings provide crucial immunomodulatory support for inhibiting tumor progression (Zhao et al., 2014).

ISL also exhibits microbiota-modulating activity. In a mouse model of colitis-associated CRC (CAC) induced by azoxymethane (AOM) and dextran sulfate sodium (DSS), ISL ameliorates gut dysbiosis, restores microbial diversity, and increases the abundance of beneficial butyrate-producing bacteria. Concurrently, it reduces the levels of opportunistic pathogens. These changes contribute to protection against CAC development and suggest a potential synergistic anticancer effect between ISL and the gut microbiota (Wu M. et al., 2016).

The study employed multiple control groups: a healthy control (CK), an ISL-only group (ISL + CK), a colitis-associated colorectal cancer CAC model (CACM) group, and ISL intervention groups at low, medium, and high doses (CIL/CIM/CIH). Crucially, the gut microbiota composition in the ISL + CK group showed no significant difference from the CK group, demonstrating that ISL alone does not alter the normal microbiota. When the high-dose ISL (CIH) group was compared to the CACM group, ISL was found to restore microbiota balance specifically within the CACM. This indicates that its beneficial effect is dependent on the presence of the dysbiotic microenvironment associated with the disease state. Furthermore, the study observed that ISL reduced pro-inflammatory cytokines and noted an enrichment of opportunistic pathogens in the CACM group. This suggests that ISL exerts its anti-cancer effects indirectly, at least partially, by suppressing such pathogenic bacteria, thereby providing indirect evidence for microbiota-host interactions. Methodologically, the researchers integrated high-throughput sequencing (16S rRNA), terminal restriction fragment length polymorphism (T-RFLP), and qPCR techniques. This comprehensive approach enabled detailed analysis of microbiota composition, diversity, and changes in the abundance of specific bacterial taxa, which were quantified alongside key anti-cancer efficacy indicators. The results revealed that high-dose ISL (150 mg/kg) significantly reduced tumor incidence. Notably, the timeline of microbiota changes closely paralleled the observed anti-cancer effects, a correlation strongly supported by significant P-values. This association was further reinforced by a clear dose-dependent effect, evident when comparing the low, medium, and high-dose ISL intervention groups. Biologically, the study proposes a mechanism whereby ISL increases the abundance of butyrate-producing bacteria, thereby promoting the generation of short-chain fatty acids (SCFAs) like butyrate. This, in turn, is hypothesized to suppress inflammatory cytokines and inhibit cancer cell growth, a connection grounded in previous research. However, the reliability of the evidence supporting this causal chain has certain limitations. Although significant correlations exist between microbiota shifts and anti-cancer outcomes, the study did not directly test the necessity of these microbiota changes through causal experiments. Additionally, it did not quantify key intermediary variables like butyrate concentration *in vivo*. Consequently, the proposed mechanism relies on indirect inference rather than direct empirical validation. Future studies should address these aspects by incorporating causal validation experiments and direct metabolite measurements, and should extend this research to relevant human tissues and cells.

**FIGURE 4:**
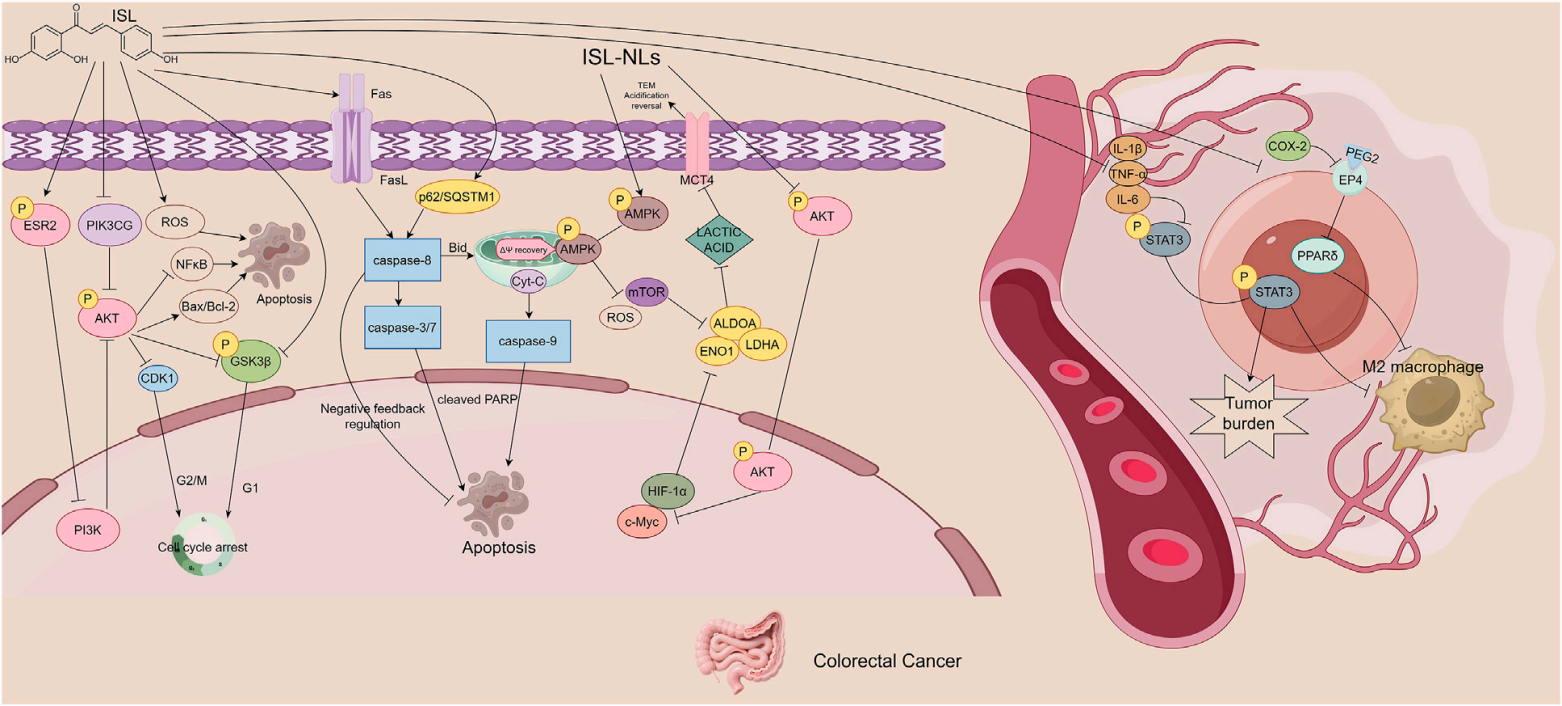
The figure illustrates the mechanisms by which ISL exerts its antitumor effects in colorectal cancer. ISL activates the ESR2/PI3K/AKT signaling axis, downregulates pro-proliferative and anti-apoptotic proteins, and upregulates pro-apoptotic proteins, thereby inhibiting cell proliferation and inducing apoptosis. It also enhances TRAIL-mediated caspase-dependent apoptosis by upregulating DR5, showing synergistic effects with chemotherapeutic agents. Additionally, ISL reduces tumor-promoting M2 macrophage polarization by suppressing pro-inflammatory mediators and related signaling pathways. ISL improves gut microbiota dysbiosis by increasing butyrate-producing beneficial bacteria and reducing opportunistic pathogens. Furthermore, ISL-loaded nanoliposomes inhibit key glycolytic enzymes and disrupt energy metabolism by regulating the AMPK/mTOR signaling pathway.

ISL-loaded nanoliposomes represent a novel drug delivery system with excellent self-assembly and biocompatibility properties. Through modulation of the AMPK/mTOR signaling pathway, ISL nanoliposomes suppress the expression of key glycolytic enzymes including Enolase 1 (ENO1), Aldolase A (ALDOA), lactate dehydrogenase A (LDHA), and monocarboxylate transporter 4 (MCT4). This results in decreased glucose uptake and lactate production, significantly inhibiting the energy metabolism and proliferation of CRC cells (Wang et al., 2020). It can be seen that intestinal flora regulation, the TRAIL/DR5 apoptotic pathway, and M2 macrophage inhibition are the specific targets of ISL in acting on colorectal tumors. By virtue of these targets, the mechanism of action of ISL in colorectal tumors is distinguished from that in other digestive system tumors (Figure 4).

### 2.4 Anticancer mechanisms of ISL in hepatocellular carcinoma

Hepatocellular carcinoma (HCC), a primary malignancy originating from hepatocytes, is one of the most prevalent tumor types encountered in clinical practice. Biologically, HCC typically presents a solid growth pattern (Allemani et al., 2018; Yang et al., 2019). Its pathogenesis involves a complex network of molecular mechanisms, among which the activation of the MAPK/STAT3/NF-κB signaling axis by ROS in the TME plays a pivotal role in regulating cell proliferation and apoptosis (Antognelli et al., 2013; Bolisetty and Jaimes, 2013).

Song et al. demonstrated that ISL induces apoptosis in HCC cells through multiple signaling pathways. *In vitro* studies revealed that ISL inhibits the viability and proliferation of HCC cell lines in a dose- and time-dependent manner. ISL significantly upregulates pro-apoptotic proteins Bax, cleaved caspase-3, and cleaved PARP, while downregulating the anti-apoptotic protein Bcl-2, indicating mitochondrial pathway-mediated apoptosis.

Moreover, ISL activates autophagy by suppressing the PI3K/AKT/mTOR signaling pathway, as evidenced by increased expression of autophagy markers Microtubule-Associated Protein 1 Light Chain 3-II (LC3-II) and Beclin 1 Autophagy Related Gene (Beclin-1). Notably, the use of autophagy inhibitors enhances ISL-induced apoptosis, suggesting a dynamic crosstalk between autophagy and apoptosis. *In vivo* experiments using a xenograft model further validated the antitumor effect of ISL: treatment with ISL (50 mg/kg) significantly suppressed tumor growth and upregulated both LC3-II and cleaved caspase-3, confirming that ISL inhibits HCC progression via synergistic induction of autophagy and apoptosis (Song et al., 2020).

ISL also inhibits HCC cell proliferation by inducing cell cycle arrest. Wang et al. reported that ISL treatment led to a marked increase in the proportion of HepG2 cells arrested at the G2/M phase, accompanied by upregulation of the cyclin-dependent kinase inhibitor p21 and downregulation of Cyclin-Dependent Kinase 1/2 (CDK1/2), Cyclin B1, and p27. This effect is closely linked to ROS accumulation: ISL, via its α, β-unsaturated carbonyl structure, depletes intracellular antioxidants resulting in elevated ROS levels. This redox imbalance activates c-Jun N-terminal Kinase (JNK) and p38 Mitogen-Activated Protein Kinase (p38 MAPK), while suppressing Extracellular Signal-Regulated Kinase (ERK), Signal Transducer and Activator of Transcription 3 (STAT3), and NF-κB pathways, ultimately triggering mitochondria-dependent apoptosis. The apoptotic effect of ISL can be reversed by the ROS scavenger N-acetylcysteine (NAC), underscoring the central role of oxidative stress in its mechanism of action (Wang et al., 2019).

**FIGURE 5:**
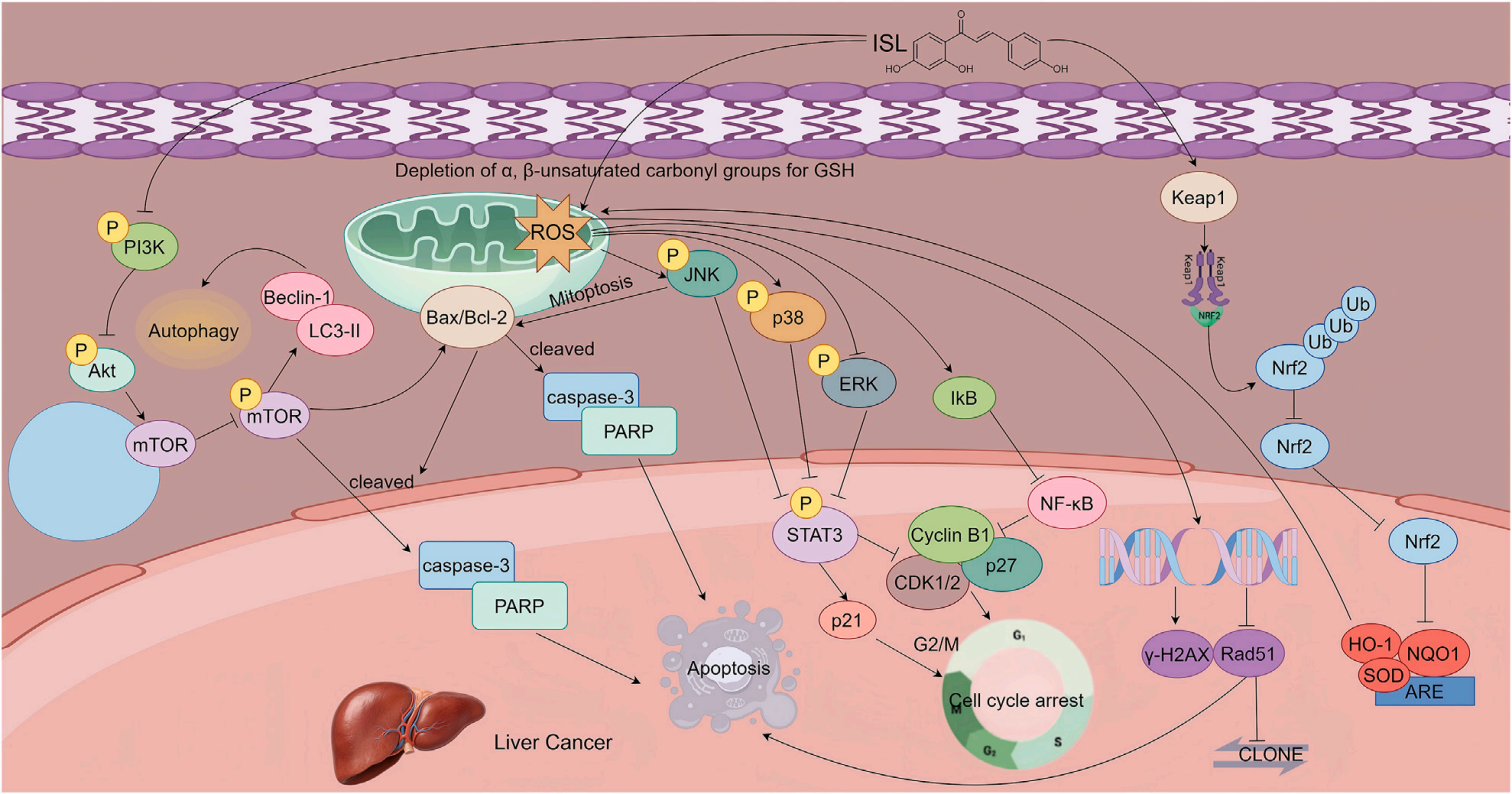
This figure illustrates the mechanisms by which ISL exerts its antitumor effects in hepatocellular carcinoma. ISL induces apoptosis in a dose -dependent manner by upregulating pro-apoptotic proteins and downregulating anti-apoptotic Bcl-2. It activates autophagy through inhibition of the PI3K/AKT/mTOR signaling pathway, which synergizes with apoptosis to suppress tumor growth. Additionally, ISL induces G2/M phase cell cycle arrest and triggers mitochondrial apoptosis by promoting ROS accumulation, which in turn activates the JNK/p38 MAPK pathways and inhibits the ERK pathway. Furthermore, ISL enhances radiosensitivity by upregulating Keap1, thereby promoting the ubiquitin-mediated degradation of Nrf2 and downregulating antioxidant gene expression.

ISL also enhances the radiosensitivity of HCC cells by modulating oxidative stress pathways. Mechanistic studies by Sun et al. showed that ISL selectively upregulates Kelch-like ECH-associated protein 1 (Keap1), promoting the ubiquitination and degradation of nuclear factor erythroid 2–related factor 2 (Nrf2), thereby inhibiting its nuclear translocation. This leads to decreased expression of downstream antioxidant genes resulting in intracellular redox imbalance. The induced pro-oxidant state sensitizes HCC cells to radiation. Pre-treatment with ISL (10 μg/mL), combined with X-ray irradiation (4 Gy), significantly increased the formation of DNA damage markers and markedly suppressed tumor growth in xenograft models. Notably, *in vivo* results revealed that the combination of ISL and radiotherapy reduced tumor volume by 80%, highlighting the potential of ISL as a radiosensitizing agent in HCC treatment (Sun et al., 2015). In hepatocellular carcinoma, ISL exerts its effects through specific mechanisms: regulating oxidative stress via the Keap1/Nrf2 pathway and selectively activating the JNK/p38 pathway. Such a mode of action is specific, serving as a distinctive feature that differentiates this tumor from other digestive system tumors (Figure 5).

### 2.5 Anticancer mechanisms of ISL in gallbladder cancer

Gallbladder carcinoma (GBC) is the most common histological subtype of primary malignancies in the biliary tract. It is characterized by highly aggressive biological behavior and limited responsiveness to current treatment modalities (Roa et al., 2022). Recent studies have highlighted the anti-cancer potential of ISL in GBC, primarily through the induction of ferroptosis—a non-apoptotic, iron-dependent form of regulated cell death. ISL exerts its effects via activation of the p62-Keap1-Nrf2-HMOX1 signaling axis and downregulation of GPX4, offering a novel therapeutic strategy for GBC.

Wang et al. demonstrated that ISL significantly inhibits the viability of GBC cell lines in a dose- and time-dependent manner. Cell Counting Kit-8 (CCK-8) assays and colony formation assays revealed that ISL markedly suppresses the clonogenic potential of GBC cells, suggesting robust anti-proliferative activity. Morphological observations further supported this conclusion: ISL-treated cells exhibited reduced numbers and increased cell volume, indicating the induction of cell death.

ISL inhibits GBC cell growth primarily by inducing ferroptosis—a unique form of programmed cell death distinct from apoptosis, necrosis, or necroptosis, characterized by iron accumulation and lipid peroxidation. Transcriptome sequencing and bioinformatics analyses revealed significant changes in ferroptosis-related genes upon ISL treatment. In particular, upregulation of heme oxygenase 1 (HMOX1) and downregulation of glutathione peroxidase 4 (GPX4) emerged as key molecular events mediating ISL-induced ferroptosis.

Mechanistically, ISL promotes ferroptosis by modulating iron metabolism and enhancing oxidative stress. ISL treatment significantly elevated intracellular ferrous ion (Fe^2+^) levels, along with a marked increase in ROS and lipid peroxidation. Additionally, ISL reduced the ratio of reduced glutathione (GSH) to oxidized glutathione (GSSG), aggravating the oxidative burden and pushing the cells toward ferroptotic death. It is important to emphasize that, in comparison with other digestive system tumors, the ferroptosis mechanism constitutes a unique mode of action for ISL in the treatment of gallbladder cancer.

Immunohistochemical analysis of xenograft tumor tissues in mice further validated these findings. Tumors derived from ISL-treated GBC models exhibited significantly increased expression of HMOX1 and reduced expression of GPX4, confirming the *in vivo* relevance of ISL-induced ferroptosis in suppressing tumor grow (Wang Z. et al., 2023).

It should be noted that the mechanism of ferroptosis mainly involves iron-dependent lipid peroxidation and ROS generation. These ROS signals can propagate among cell populations through the so-called “trigger waves,” leading to oxidative damage and even death of adjacent non-tumor cells (Co et al., 2024). In addition, studies have shown that the activation of ferroptosis may cause functional damage to healthy tissues (Zhang C. et al., 2022; Chen et al., 2024). Meanwhile, the ROS leakage and release of damage-associated molecular patterns (DAMPs) accompanied by ferroptosis can damage immune cells, resulting in immunosuppression or pro-inflammatory responses, thereby weakening the therapeutic effect and even affecting the effectiveness of combined immunotherapy (Zhou et al., 2024; Qi and Peng, 2023).

**FIGURE 6:**
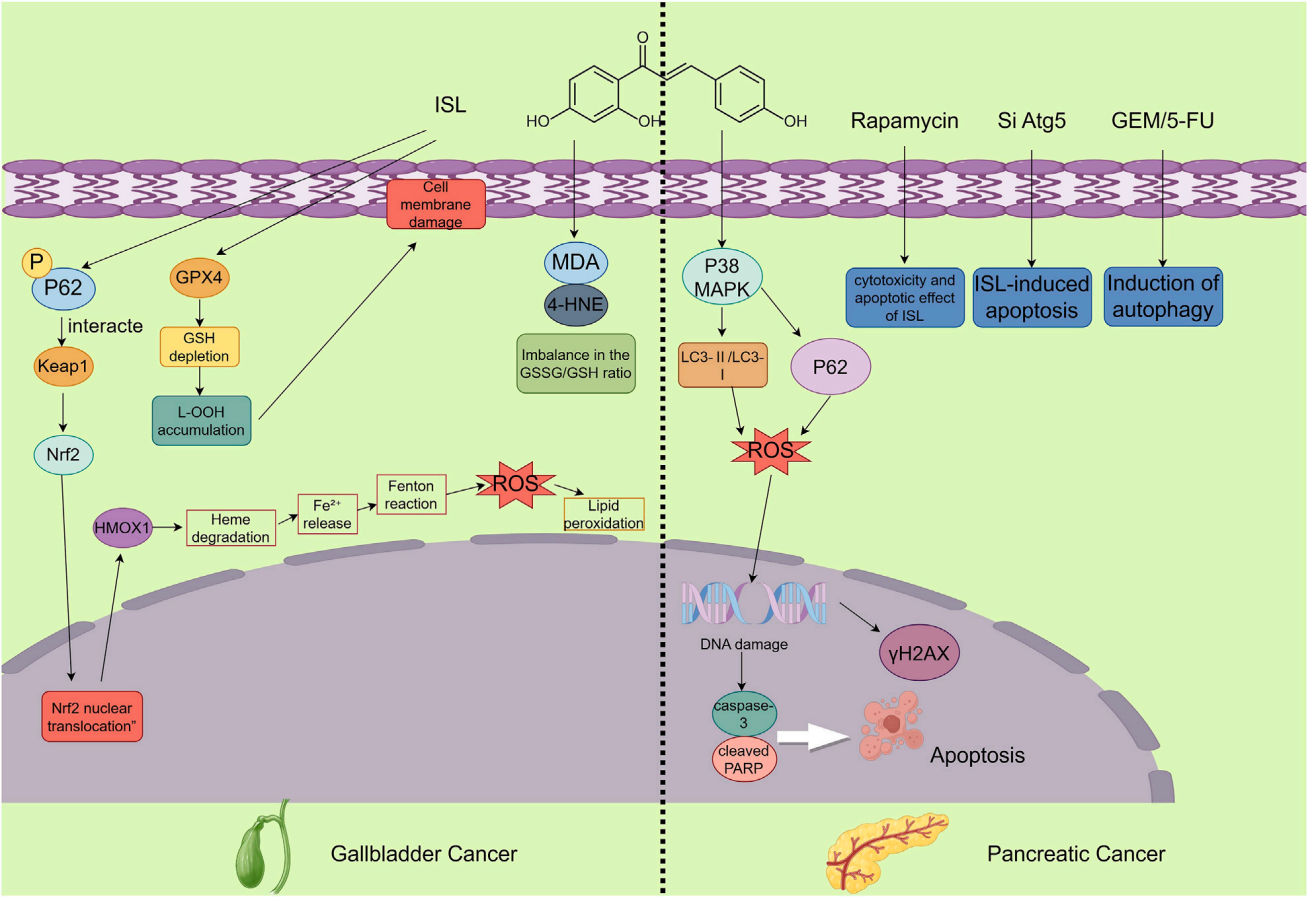
The figure illustrates the mechanisms by which ISL exerts its antitumor effects in gallbladder and pancreatic cancers. In gallbladder cancer, ISL demonstrates anticancer potential by inducing ferroptosis. This involves activation of the p62-Keap1-Nrf2-HMOX1 signaling axis and downregulation of GPX4, accompanied by increased intracellular Fe^2+^, ROS, and lipid peroxidation levels, along with a decreased GSH/GSSG ratio, ultimately inhibiting the proliferation of GBC cells. In pancreatic cancer, ISL promotes apoptosis and blocks autophagic flux—evidenced by the accumulation of LC3-II and p62—through activation of the p38 MAPK signaling pathway. Moreover, ISL enhances the cytotoxic effects of chemotherapeutic agents and significantly suppresses tumor growth *in vivo*.

To mitigate these risks, we can use antioxidants to slow down the excessive reaction of ferroptosis (Chen et al., 2024), as well as iron chelators or iron-removing drugs to reduce cell damage caused by iron overload and protect normal tissues from oxidative stress (Zhang C. et al., 2022; Chen et al., 2024). Besides, the use of targeted drug delivery systems can directionally deliver ferroptosis inducers to tumor sites instead of systemic administration, which can alleviate oxidative stress responses in healthy tissues (Dang et al., 2022). Furthermore, in clinical treatment, ferroptosis can be mitigated through individualized treatment regimens and dosage adjustments (Zhou et al., 2024; Qi and Peng, 2023) (Figure 6).

### 2.6 Anticancer mechanisms of ISL in pancreatic cancer

Pancreatic cancer, particularly pancreatic ductal adenocarcinoma (PDAC), is a highly lethal malignancy characterized by poor prognosis, early metastasis, and limited responsiveness to conventional therapies (Wood et al., 2022). A defining feature of PDAC is its intrinsically high basal autophagy activity, which contributes to chemoresistance and tumor progression (Vincent et al., 2011). Therefore, identifying novel agents that can modulate autophagy and enhance chemotherapy efficacy is of urgent clinical importance. Recent studies suggest that ISL exhibits promising anti-tumor activity against pancreatic cancer, primarily by disrupting autophagy through modulation of the p38 MAPK signaling pathway.

Zhang et al. demonstrated that ISL inhibits autophagic flux in pancreatic cancer cells by activating the p38 MAPK signaling pathway, thereby inducing cell apoptosis and suppressing tumor growth. Specifically, ISL leads to the accumulation of autophagosome markers LC3-II and p62, while blocking the fusion of autophagosomes with lysosomes. When combined with gemcitabine (GEM) and 5-fluorouracil (5-FU), ISL enhances the cytotoxicity of these chemotherapeutic agents, resulting in a synergistic anti-tumor effect. In *in vivo* experiments, ISL significantly inhibited tumor growth in mouse models of pancreatic cancer and increased the number of apoptotic cells in tumor tissues. These findings collectively highlight the pharmacological potential of ISL as an adjuvant therapeutic agent for PDAC, acting through modulation of p38 MAPK-dependent autophagy pathways (Zhang et al., 2022c). Meanwhile, this mechanism also represents the unique feature that distinguishes pancreatic cancer from other digestive system tumors in terms of ISL treatment (Figure 6).

### 2.7 Delivery challenges and optimization strategies of nanoscale delivery systems in anticancer applications of ISL

One of the major challenges in the clinical application of ISL as a candidate anticancer agent lies in its poor aqueous solubility, which directly hinders the development of effective drug delivery systems (Yamamoto et al., 1991). Although studies on ISL’s bioavailability and pharmacokinetic parameters have achieved preliminary progress, its physicochemical limitations remain a bottleneck. To address this issue, nanostructured lipid carriers (NLCs), composed of a mixture of solid lipids and liquid lipids, have been developed. These NLCs form spherical particles with a mean diameter of 160.73 nm and a high encapsulation efficiency of 96.74%. The hydrophobic lipid core effectively entraps ISL, while the hydrophilic outer shell, formed by Poloxamer 188 and Tween 80, enhances dispersion stability (Yamamoto et al., 1991). *In vitro* release studies have shown that ISL-NLC exhibits a biphasic release profile, with 57.76% of ISL released within 8 h and sustained release up to 24 h, conforming to the Weibull kinetic model. This dual release behavior supports both rapid onset and prolonged activity (Sung et al., 2021). In tumor-bearing mice, ISL-NLC improved ISL accumulation in tumor tissue by 2.5-fold via the enhanced permeability and retention (EPR) effect, with the area under the curve (AUC) reaching 19.04 mg/mL·h and the half-life (t_1_/_2_) extended to 7.26 h. Lymphatic absorption further reduced hepatic first-pass metabolism (Yamamoto et al., 1991; Wang et al., 2012). These advantages are consistent with the general benefits of lipid-based delivery systems: lipid nanoparticles can passively target tumors through the EPR effect and reduce clearance by the reticuloendothelial system (RES) via surface modifications, thereby prolonging systemic circulation (Allen and Cullis, 2013). Efforts to enhance delivery efficiency through active targeting have also been explored. For example, Gao et al. developed iRGD-modified lipid–polymer hybrid nanoparticles (ISL-iRGD NPs), with a particle size of 137.2 nm and an encapsulation efficiency of 90.8%. The iRGD peptide specifically binds to integrin αvβ3 and neuropilin-1 (Nrp1) receptors overexpressed on tumor cells, leading to a 2.3-fold increase in cellular uptake in MDA-MB231 breast cancer cells. In a 4T1 tumor-bearing mouse model, these nanoparticles significantly enhanced tumor inhibition through combined active targeting and EPR effects (Gao et al., 2017). Similarly, Wang et al. used liposomal delivery of quercetin, and PEGylation extended the circulation half-life by threefold and improved AUC by 2.1-fold, validating the stealth and tumor-accumulating properties of lipid-based systems (Gang et al., 2012). Lipid–polymer hybrid nanoparticles integrate the biocompatibility of lipids with the mechanical stability of polymers and have demonstrated superior lymphatic targeting efficiency in gastric cancer models (Yang et al., 2013). Although ISL-NLC formulations exhibit good stability under lyophilization (protected with 5% lactose or glucose, with no aggregation after 6 months) and manageable toxicity (body weight loss <10%) (Zhang et al., 2013), future studies should apply UPLC-MS/MS techniques to precisely elucidate ISL’s metabolic dynamics (Wang et al., 2012). Furthermore, incorporating tumor-specific ligands into targeted nanocarriers tailored for gastrointestinal malignancies holds great promise (Gao et al., 2017). Notably, NLCs outperform traditional solid lipid nanoparticles (SLNs) by reducing crystallinity through the combination of solid and liquid lipids. This design improves drug loading capacity by 12%–15% and reduces drug leakage during storage by approximately 40%, making NLCs particularly suitable for high-dose sustained-release formulations (Pardeike et al., 2009) In an H22 HCC model, ISL-NLC significantly inhibited tumor growth via upregulation of the p53/p21 pathway, induction of G2/M phase arrest, and inhibition of topoisomerase II activity. At a dose of 40 mg/kg, the tumor inhibition rate reached 85.62%, indicating a dose-dependent antitumor effect that is closely associated with enhanced intratumoral drug accumulation enabled by lipid-based nanocarriers (Zhang et al., 2013; Wang et al., 2012).

Pharmacokinetic optimization via nanocarrier systems represents an effective strategy to enhance the bioavailability of ISL. Polymer micelle-based systems have shown promise in significantly improving ISL’s aqueous solubility—up to 232-fold—by encapsulating ISL within their hydrophobic cores. In simulated gastrointestinal conditions, these micelles achieved over 80% cumulative drug release. Pharmacokinetic studies demonstrated a 2.23-fold increase in bioavailability compared to free ISL, along with an extended plasma half-life of 4.71 h (Xie et al., 2019). Hybrid membrane nanoparticles formed by fusing red blood cell and tumor cell membranes—have further enhanced ISL delivery. These biomimetic nanoparticles retain critical membrane proteins enabling both long-circulation capability and homotypic targeting. With an encapsulation efficiency of 55.6%, ISL@HM NPs induced apoptosis (84.4%) and cellular senescence (82.9%) *in vitro*, primarily through activation of the mitochondrial apoptotic pathway (Shi et al., 2022). In addition, pH-responsive micellar systems, exploit the acidic TME to achieve site-specific drug release. These micelles released approximately 90% of ISL under acidic conditions, while drug release in physiological pH remained limited to 60%. Pharmacokinetic profiling showed a 2.6-fold increase in plasma half-life and a 2.58-fold improvement in tumor accumulation compared to non-targeted formulations (Song et al., 2022). This strategy, leveraging both EPR effect and pH-responsiveness, is particularly well-suited for targeting gastrointestinal tumors, where localized acidity and vascular abnormalities are common.

Optimization of ISL delivery via two advanced nanocarrier systems has significantly addressed its poor aqueous solubility and limited bioavailability. Zhang et al. developed a self-microemulsifying drug delivery system (SMEDDS) composed of ethyl oleate, Tween 80, and PEG 400. This formulation generated nanoscale droplets (44.78 ± 0.35 nm) with a high encapsulation efficiency of 98.17%, markedly enhancing ISL’s solubility and oral bioavailability. Under simulated gastrointestinal conditions, the 24-h cumulative release of ISL from SMEDDS was 78.95% in HCl solution—1.5-fold higher than that of free ISL (51.08%). Furthermore, SMEDDS significantly increased the AUC_0_–_24_h from 2.72 μg·h/mL to 12.81 μg·h/mL, corresponding to a 4.71-fold enhancement in bioavailability, likely via P-gp inhibition or lymphatic transport. The formulation remained stable over 3 months under accelerated storage, with droplet size increasing only slightly to 50.31 nm, and exhibited good stability at room temperature for at least 30 days (Zhang et al., 2019). In another study, a nanoemulsion (ILQ-NE) was fabricated using a sonication-phase inversion composition (SPIC) method with Labrafil^®^ M 1944 CS as the oil phase and Cremophor^®^ EL as the surfactant. ILQ-NE achieved a droplet size of 44.10 ± 0.28 nm and a narrow polydispersity index (PDI = 0.098). It improved ISL solubility to 4 mg/mL—approximately 1,000-fold higher than its inherent aqueous solubility (3.74 μg/mL). The formulation exhibited sustained release, with a 36-h cumulative release of 78.43%, compared to 50.30% for free ISL. After 56 days at 4 °C, no significant change in droplet size was observed, and ISL retained over 80% stability under UV exposure (vs. <50% for free ISL). Notably, ILQ-NE exhibited enhanced cytotoxicity against 4T1 breast cancer cells (only 30% viability at 20 μg/mL) and significantly increased cellular uptake, with a fluorescence intensity 9.38 times higher than that of free ISL, attributed to improved endocytosis via the small droplet size (Wang et al., 2022). Each system offers unique advantages: SMEDDS provides higher drug loading (7.69% vs. 4%), making it suitable for oral delivery and treatment of metabolic diseases, whereas ILQ-NE avoids thermal degradation of heat-sensitive compounds and demonstrates superior anticancer efficacy via the enhanced EPR effect. Future studies may explore hybrid strategies that combine the lymphatic transport benefits of SMEDDS with the tumor-targeting capabilities of ILQ-NE. Surface modifications could further improve tumor selectivity and therapeutic outcomes (Table 2).

**TABLE 2:** Pharmacokinetic parameters of ISL and ISL preparations.

Drug	Half-life	AUC_0–24h_	${\text{AUC}}_{0-}\infty$	AUC_0–36h_	Relative bioavailability (compared with ISL)	Notes	References
Free ISL	0.70 ± 0.22 h (Intravenous injection, SD rats)	12.61 ± 2.47 μg·h·mL^−1^ (Oral administration, SD rats)	372.75 ± 105.57 ng/mL·h (Intravenous injection, SD rats)	2.72 ± 0.26 μg/mL (Oral administration, SD rats)	NA	NA	Zhang et al. (2013), Xie et al. (2019), Zhang et al. (2019)
ISL-FPM	4.71 ± 1.02 h	NA	555.25 ± 60.42 ng/mL·h	NA	148.96%	Intravenous injection, SD rats	Zhang et al. (2013)
ISL-NLC	7.26 ± 0.14 h (Plasma) 9.17 ± 0.18 h (Tumor)	NA	19.04 ± 1.25 μg/mL/h (Plasma) 4.73 ± 0.19 μg/mL/h (Tumor)	NA	approximately 250%	Intraperitoneal injection, H22 tumor - bearing mice	Zhang et al. (2013)
ISL-M	1.82 ± 0.35 h	NA	555.25 ± 60.42 ng/mL·h	NA	148.96%	Intravenous injection, SD rats	Zhang et al. (2013)
ISL-SMEDDS	24.07 ± 0.83 h	NA	NA	12.81 ± 1.40 μg/mL	471%	Oral administration, SD rats	Zhang et al. (2019)

## 3 Discussion

**FIGURE 7:**
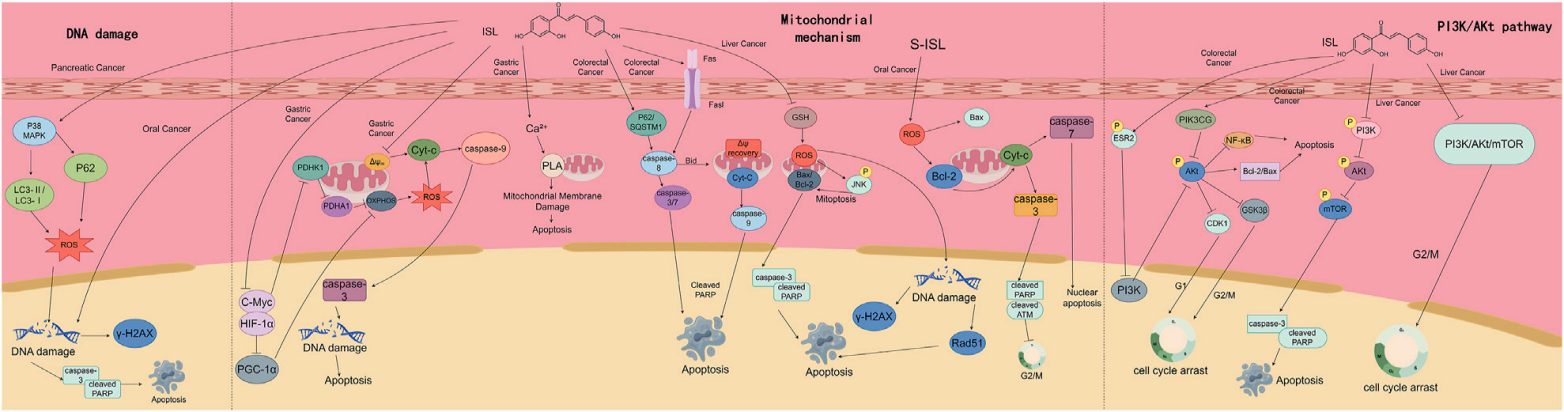
This figure demonstrates the mechanisms by which ISL exerts therapeutic effects on multiple digestive system tumors. It presents three main pathways: the DNA damage - inducing pathway, the mitochondria - mediated apoptosis pathway, and the regulatory mechanism of the PI3K/Akt pathway. Through these pathways, ISL triggers processes such as DNA damage, mitochondrial membrane disruption, caspase activation, and cell cycle arrest, ultimately leading to tumor cell apoptosis, and thus plays a role in the treatment of various digestive system tumors including pancreatic cancer, gastric cancer, colorectal cancer, and liver cancer.

Gastrointestinal cancers, including colorectal, gastric, hepatic, esophageal, and pancreatic cancers, represent a major global cancer burden. According to the 2008 global cancer statistics, these five types of malignancies accounted for approximately 29.30% of all newly diagnosed cancer cases and were responsible for 35.86% of cancer-related deaths worldwide (Kobayashi et al., 2019). The high mortality rate associated with gastrointestinal tumors is largely attributed to chemotherapy resistance and frequent recurrence, which are driven by complex oncogenic mechanisms involving both intrinsic tumor cell properties and interactions within the TME (Gong et al., 2014). ISL is a natural flavonoid compound primarily extracted from medicinal plants such as *Glycyrrhiza uralensis*, *Sinofranchetia chinensis*, and *Dalbergia odorifera* (Huang X. et al., 2020). ISL exhibits a broad spectrum of pharmacological activities. Accumulating evidence has demonstrated its anti-tumor potential through multiple mechanisms, including induction of cell cycle arrest, suppression of glycolytic metabolism, inhibition of proliferation and migration, and promotion of apoptosis—particularly in HCC and other digestive system tumors. However, current research on ISL remains largely confined to *in vitro* cell-based assays and *in vivo* animal models. Clinical studies are lacking, making it difficult to comprehensively assess its therapeutic efficacy and safety in humans. Furthermore, like many flavonoids, ISL suffers from poor oral bioavailability and rapid metabolic clearance. Its pharmacokinetics—including tissue distribution, metabolic pathways, and effective systemic concentrations—have not been fully elucidated and warrant further investigation. Meanwhile, comparative studies have confirmed that ISL exhibits unique mechanisms of action that have been validated by research. These include the induction of ferroptosis in gallbladder cancer via downregulation of GPX4 through the p62-Keap1-Nrf2-HMOX1 axis, as well as the promotion of Bax/Bcl-2 alterations, activation of caspases, and induction of G2/M phase arrest through mitochondrial pathways in colorectal cancer models. In contrast, other mechanisms (Yoshida et al., 2008), autophagy-apoptosis crosstalk (Huang Z. et al., 2020), and reversal of drug resistance (Kim et al., 2020) are commonly observed across various flavonoids. In general, alongside these unique mechanisms specific to certain tumors, ISL also employs some therapeutic mechanisms that are shared across multiple types of digestive system tumors. Among them, ISL commonly causes cell cycle arrest and apoptosis in digestive system tumor cells through DNA damage, mitochondrial mechanisms, and the PI3K/AKT pathway (Figure 7).

ISL shows potential in diverse cancers, with overlapping effects in digestive and other malignancies: inducing apoptosis (Wang et al., 2019); via Caspase in cervical cancer (Wu C.-H. et al., 2016) and regulating PI3K/Akt (autophagy in pancreatic cancer) (Zhang et al., 2022c); breast cancer proliferation (Xie et al., 2025). And digestive tumor research can reference its mechanisms elsewhere, e.g., metastasis inhibition via circNAV3/miR-4262/ST6GALNAC5/EGFR (as in triple-negative breast cancer) (Xie et al., 2025) or progression suppression through m6A/IGF2BP3-stabilized TWIST1 mRNA (Cui et al., 2022).

At the same time research on the therapeutic role of ISL in digestive system tumors can draw inspiration from its therapeutic mechanisms in other types of tumors to conduct in-depth mechanistic exploration. For instance, it can be investigated whether ISL can inhibit the metastatic progression of digestive system tumors by regulating the circNAV3/miR-4262/ST6GALNAC5/EGFR axis, which is analogous to ISL’s role in triple-negative breast cancer (Xie et al., 2025). Additionally, research can explore whether ISL suppresses the progression of digestive system tumors through m6A/IGF2BP3-mediated stabilization of TWIST1 mRNA (Cui et al., 2022), and so on.

ISL formulations have not entered early clinical trials for two reasons: metabolic uncertainties and interspecies differences (ISL has high absorption but low bioavailability in rats (Lee et al., 2013), making animal parameters inapplicable to humans); and insufficient quality control and stability (nanoformulations lack evaluation, e.g., ISL-LP stability unestablished (Song et al., 2024), impeding translation).

Future research on ISL should focus on overcoming the major barriers that limit its clinical application. Structural modification strategies, such as the introduction of hydrophilic groups or the design of prodrug forms, can improve its physicochemical properties, enhance aqueous solubility, and increase metabolic stability, thereby addressing its poor oral absorption. In parallel, the development of novel drug delivery systems offers promising avenues to enhance tumor-specific accumulation of ISL while reducing systemic toxicity to healthy tissues. In addition, a deeper understanding of ISL’s molecular targets involved in regulating tumor immune evasion, metabolic reprogramming, and non-apoptotic forms of cell death will provide a theoretical basis for designing multi-target combination therapies. Moreover, the integration of computer-aided drug design (CADD), structure-activity relationship (SAR) modeling, and high-throughput compound screening can facilitate the rational synthesis of novel ISL derivatives with improved anticancer efficacy and reduced resistance potential. By aligning these strategies with clinical needs, ISL has the potential to overcome the conventional limitations of natural compounds and evolve into a next-generation anticancer candidate that offers both enhanced therapeutic efficacy and favorable pharmacokinetic properties. This could provide innovative solutions for the treatment of solid tumors and drug-resistant cancers. While committing to deepening research on the anticancer mechanism of ISL, efforts should be synchronously made to advance the optimization and translational research of its drug delivery systems. This is aimed at promoting the clinical application transformation of this natural active ingredient and providing stronger support for the cause of human tumor prevention and treatment.

## Glossary

ALDOA: Aldolase A
AKT: Protein Kinase B
AOM: azoxymethane
ATM: ataxia telangiectasia mutated
AUC: area under the curve
Bax: Bcl-2-associated X protein
Bcl-2: B-cell lymphoma 2
Beclin-1: Beclin 1 Autophagy Related Gene
CAC: colitis-associated
CADD: computer-aided drug design
CAF: cancer-associated fibroblast
CAFs: cancer-associated fibroblasts
CAL-27: Centre Antoine Lacassagne-27
CCK-8: Cell Counting Kit-8
CDK1: Cyclin-Dependent Kinase 1
CDK1/2: Cyclin-Dependent Kinase 1/2
c-Myc: cellular Myc
CRC: Colorectal cancer
DAMPs: damage-associated molecular patterns
DR5: death receptor 5
DSS: dextran sulfate sodium
ENO1: Enolase 1
EPR: enhanced permeability and retention
ERK: Extracellular Signal-Regulated Kinase
ESR2: Estrogen Receptor 2
GA: glycyrrhetinic acid
GBC: gallbladder carcinoma
GEM: gemcitabine
GLD: glycyrrhizic acid
GPX4: glutathione peroxidase 4
GLUT4: Glucose Transporter 4
GRP78: glucose-regulated protein 78
GSH: glutathione
GSSG: oxidized glutathione
HCC: Hepatocellular carcinoma
HIF-1α: Hypoxia-Inducible Factor-1 Alpha
HMOX1: heme oxygenase 1
HPV: human papillomavirus
IL-6: interleukin-6
ISL: isoliquiritigenin
ISL-iRGD NPs: iRGD-modified lipid–polymer hybrid nanoparticles
JNK: c-Jun N-terminal Kinase
Keap1: Kelch-like ECH-associated protein 1
LCA: licochalcone A
LCB: licochalcone B
LC3-II: Microtubule-Associated Protein 1 Light Chain 3-II
LDHA: lactate dehydrogenase A
MCT4: monocarboxylate transporter 4
MMP-2: Matrix Metalloproteinase-2
MMP-9: Matrix Metalloproteinase-9
NAC: acetylcysteine
NF-κB: Nuclear Factor kappa-light-chain-enhancer of activated B cells
NLCs: nanostructured lipid carriers
Nrf2: factor erythroid 2–related factor 2
Nrp1: neuropilin-1
OSCC-CSCs: oral squamous cell carcinoma cancer stem cells
OXPHOS: oxidative phosphorylation
PDAC: pancreatic ductal adenocarcinoma
PDHK1: Pyruvate Dehydrogenase Kinase 1
PGC-1α: Peroxisome Proliferator-Activated Receptor Gamma Coactivator 1 Alpha
PGE2: prostaglandin E2
p-GSK3β: phosphorylated Glycogen Synthase Kinase 3β
PIK3CG: Phosphatidylinositol-4,5-Bisphosphate 3-Kinase Catalytic Subunit Gamma
p38 MAPK: p38 Mitogen-Activated Protein Kinase
RES: reticuloendothelial system
ROS: reactive oxygen species
SAR: structure-activity relationship
SCC: squamous cell carcinoma
SCFAs: short-chain fatty acids
SLNs: solid lipid nanoparticles
SMEDDS: self-microemulsifying drug delivery system
SMEDDSs: self-microemulsifying drug delivery systems
STAT3: Signal Transducer and Activator of Transcription 3
TME: tumor microenvironment
T-RFLP: terminal restriction fragment length polymorphism
TRAIL: tumor necrosis factor-related apoptosis-inducing ligand
ΔΨm: mitochondrial membrane potential
5-FU: 5-fluorouracil
11-DOGA: 11-deoxoglycyrrhetinic acid
