# Correction: Targeting digestive system cancers with isoliquiritigenin: a comprehensive review of antitumor mechanisms

**DOI:** 10.3389/fphar.2025.1705251

**Published:** 2025-10-22

**Authors:** Zhichun Li, Ruohan Feng, Jinze Li, Jinglu Bai, Nuo Li, Zhenhua Cui, Xiaobin Zhang

**Affiliations:** 1 Medical College, Shandong University of Traditional Chinese Medicine, Jinan, Shandong, China; 2 College of Pharmacy, Shandong University of Traditional Chinese Medicine, Jinan, Shandong, China; 3 College of Traditional Chinese Medicine, Shandong University of Traditional Chinese Medicine, Jinan, Shandong, China; 4 Department of Colorectal and Anal Surgery, The Second Hospital of Shandong University, Jinan, Shandong, China; 5 Department of Traditional Chinese Medicine External Treatment Center, Affiliated Hospital of Shandong University of Traditional Chinese Medicine, Jinan, Shandong, China

**Keywords:** isoliquiritigenin, digestive system cancers, licorice, mechanism, natural compounds

The **Figures** were in the wrong order in the PDF and HTML version of this paper. Figure 2 was published as Figure 3, Figure 3 was published as Figure 4, Figure 4 was published as Figure 6, Figure 5 was published as Figure 2, and Figure 6 was published as Figure 5. The order has now been corrected.

**FIGURE 1:**
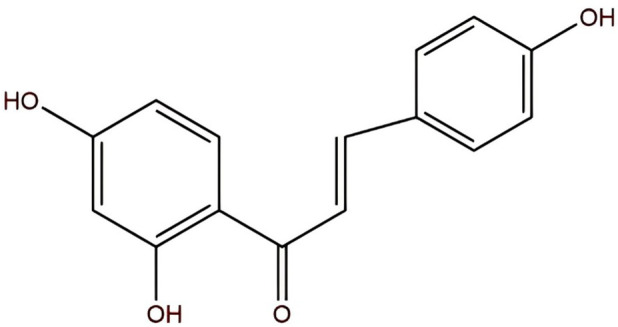
The chemical formula of ISL is C_15_H_12_O_4_.

**FIGURE 2:**
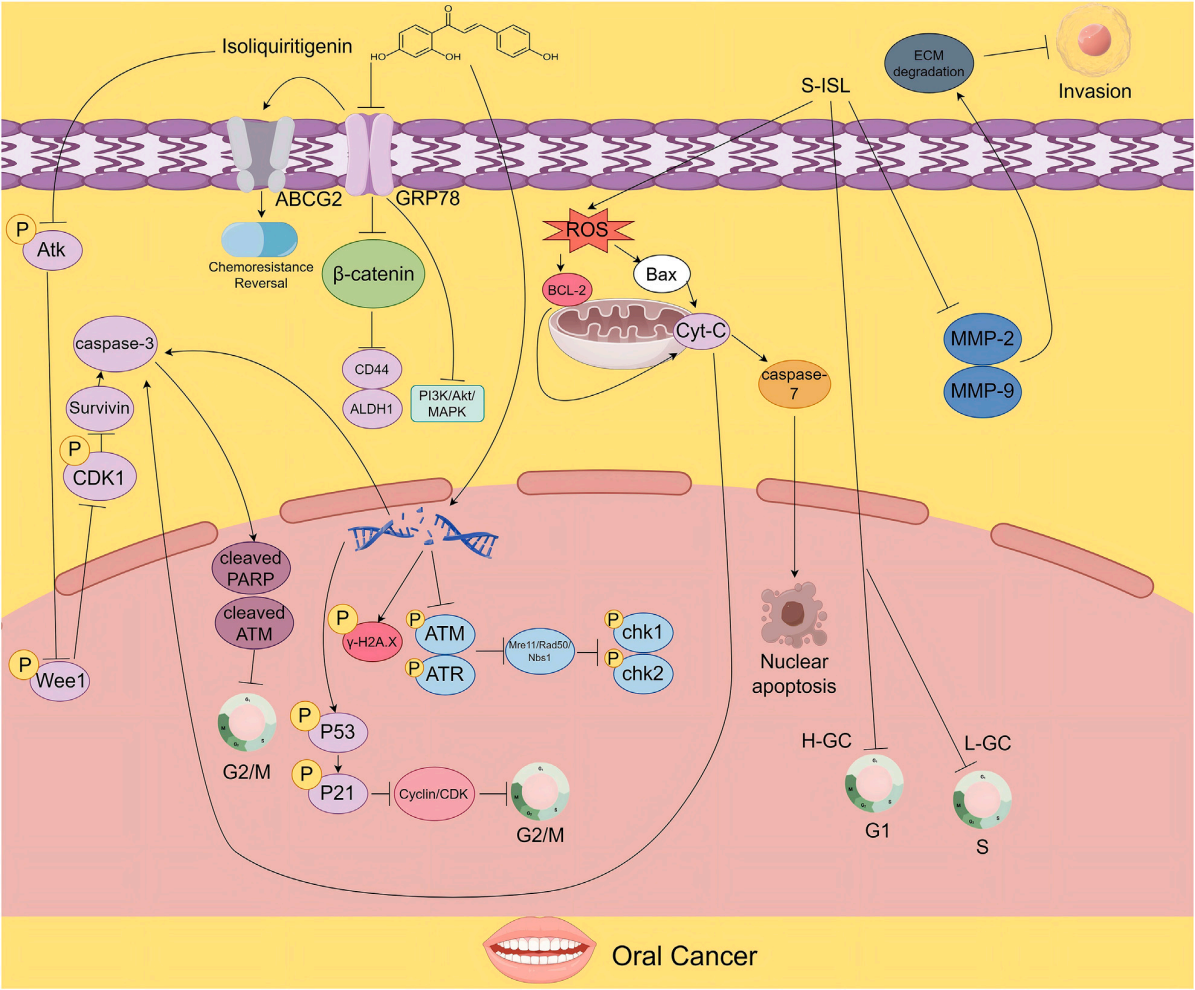
The figure illustrates the proposed mechanisms by which ISL exerts its antitumor effects in oral cancer. ISL promotes survivin degradation by inhibiting the Akt-Wee1-CDK1 signaling pathway. It also suppresses tumor growth and enhances chemosensitivity by downregulating drug resistance - associated proteins. Moreover, its derivative S-ISL exhibits antitumor activity by modulating apoptosis- and metastasis-related proteins, including Bcl-2 and Bax, and by reducing ROS production. In addition, ISL can disrupt DNA repair mechanisms, inducing cell cycle arrest and apoptosis, thereby inhibiting tumor proliferation.

**FIGURE 3:**
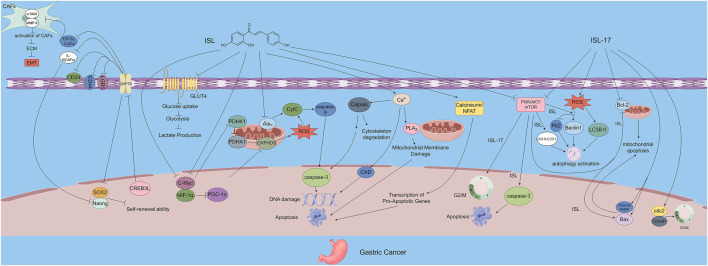
The figure illustrates the mechanisms by which ISL exerts its antitumor effects in gastric cancer. ISL-17, a synthetic derivative of ISL, inhibits tumor proliferation by inducing G2/M cell cycle arrest, promoting apoptosis, increasing ROS production, and enhancing autophagy. ISL downregulates GRP78 expression and inhibits the PI3K/AKT/mTOR signaling pathway, thereby modulating the TME, suppressing the self-renewal capacity of gastric cancer stem cells, and inactivating CAFs. In addition, ISL impairs energy metabolism by inhibiting GLUT4-mediated glucose uptake and interfering with both OXPHOS and glycolysis. Through the PDHK1/PGC-1α axis, ISL induces energy collapse and ROS accumulation, highlighting its multi-targeted antitumor potential.

**FIGURE 4:**
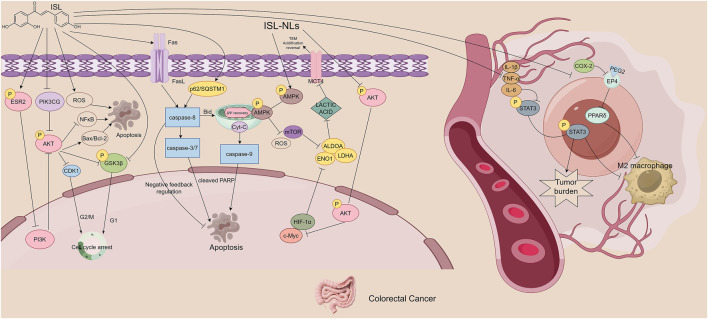
The figure illustrates the mechanisms by which ISL exerts its antitumor effects in colorectal cancer. ISL activates the ESR2/PI3K/AKT signaling axis, downregulates pro-proliferative and anti-apoptotic proteins, and upregulates pro-apoptotic proteins, thereby inhibiting cell proliferation and inducing apoptosis. It also enhances TRAIL-mediated caspase-dependent apoptosis by upregulating DR5, showing synergistic effects with chemotherapeutic agents. Additionally, ISL reduces tumor-promoting M2 macrophage polarization by suppressing pro-inflammatory mediators and related signaling pathways. ISL improves gut microbiota dysbiosis by increasing butyrate-producing beneficial bacteria and reducing opportunistic pathogens. Furthermore, ISL-loaded nanoliposomes inhibit key glycolytic enzymes and disrupt energy metabolism by regulating the AMPK/mTOR signaling pathway.

**FIGURE 5:**
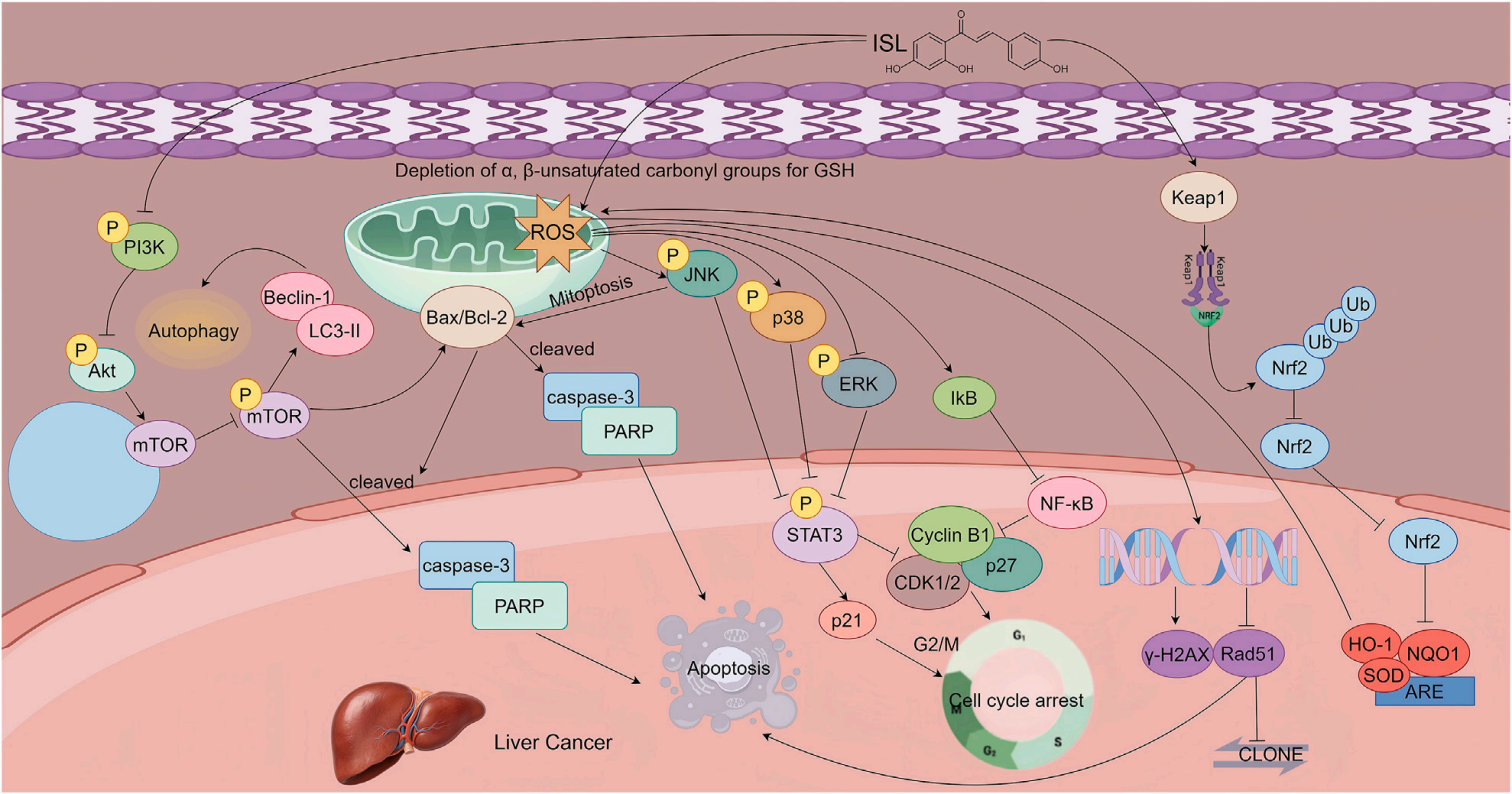
This figure illustrates the mechanisms by which ISL exerts its antitumor effects in hepatocellular carcinoma. ISL induces apoptosis in a dose -dependent manner by upregulating pro-apoptotic proteins and downregulating anti-apoptotic Bcl-2. It activates autophagy through inhibition of the PI3K/AKT/mTOR signaling pathway, which synergizes with apoptosis to suppress tumor growth. Additionally, ISL induces G2/M phase cell cycle arrest and triggers mitochondrial apoptosis by promoting ROS accumulation, which in turn activates the JNK/p38 MAPK pathways and inhibits the ERK pathway. Furthermore, ISL enhances radiosensitivity by upregulating Keap1, thereby promoting the ubiquitin-mediated degradation of Nrf2 and downregulating antioxidant gene expression.

**FIGURE 6:**
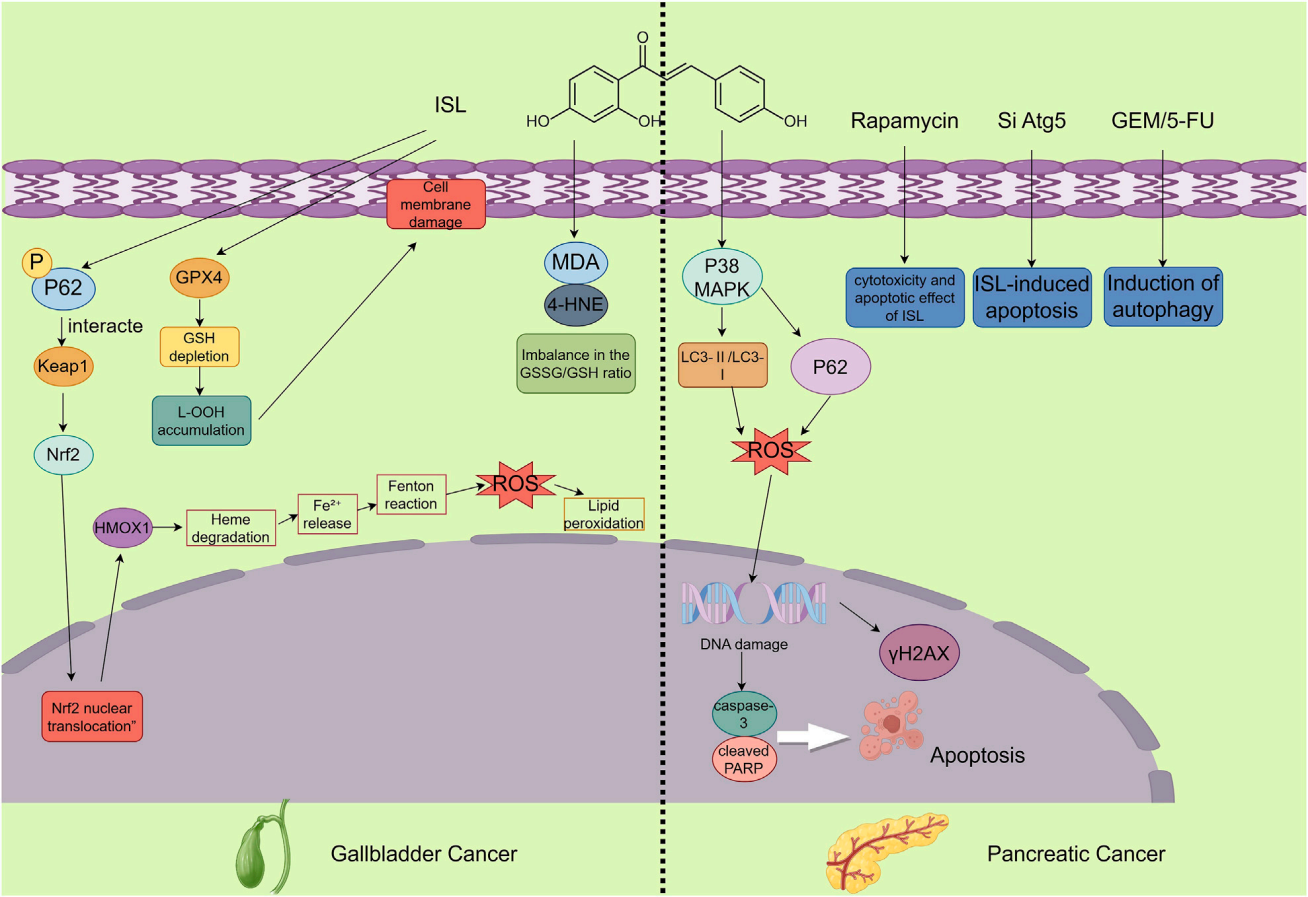
The figure illustrates the mechanisms by which ISL exerts its antitumor effects in gallbladder and pancreatic cancers. In gallbladder cancer, ISL demonstrates anticancer potential by inducing ferroptosis. This involves activation of the p62-Keap1-Nrf2-HMOX1 signaling axis and downregulation of GPX4, accompanied by increased intracellular Fe^2+^, ROS, and lipid peroxidation levels, along with a decreased GSH/GSSG ratio, ultimately inhibiting the proliferation of GBC cells. In pancreatic cancer, ISL promotes apoptosis and blocks autophagic flux—evidenced by the accumulation of LC3-II and p62—through activation of the p38 MAPK signaling pathway. Moreover, ISL enhances the cytotoxic effects of chemotherapeutic agents and significantly suppresses tumor growth in vivo.

**FIGURE 7:**
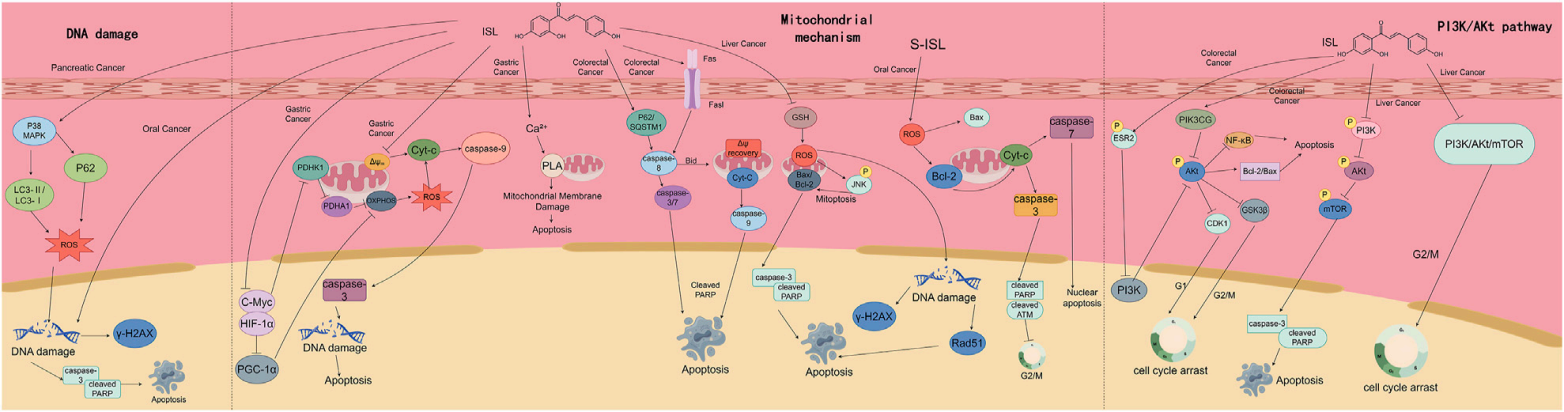
This figure demonstrates the mechanisms by which ISL exerts therapeutic effects on multiple digestive system tumors. It presents three main pathways: the DNA damage - inducing pathway, the mitochondria - mediated apoptosis pathway, and the regulatory mechanism of the PI3K/Akt pathway. Through these pathways, ISL triggers processes such as DNA damage, mitochondrial membrane disruption, caspase activation, and cell cycle arrest, ultimately leading to tumor cell apoptosis, and thus plays a role in the treatment of various digestive system tumors including pancreatic cancer, gastric cancer, colorectal cancer, and liver cancer.

The original article has been updated.

